# Supplementary material for: Photocatalytic Self-Cleaning Cotton Fabrics Coated by Cu2(OH)PO4 under VIS/NIR Irradiation
Source: Materials (Basel). 2019 Jan 11;12(2):238. doi: 10.3390/ma12020238 (PMC6356686; doi:10.3390/ma12020238)
Supplement: Supplementary file 1 [file materials-12-00238-s001.pdf]

Supplementary Materials: Photocatalytic Self-Cleaning Cotton Fabrics Coated by $Cu_2(OH)PO_4$ under Vis/NIR Irradiation

Dawei Gao, Lili Wang, Chunxia Wang and Tan Chen

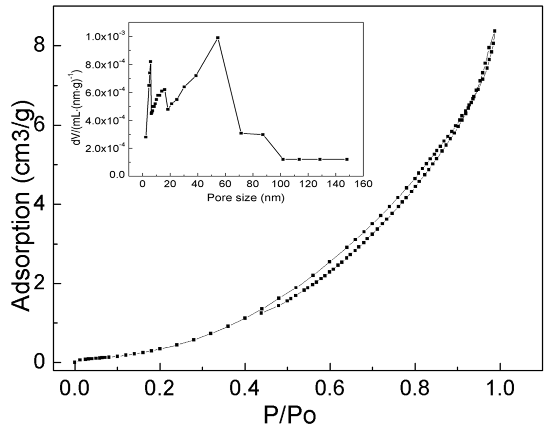

Figure S1. Nitrogen adsorption-desorption isotherm and pore size distribution of cotton fabrics.

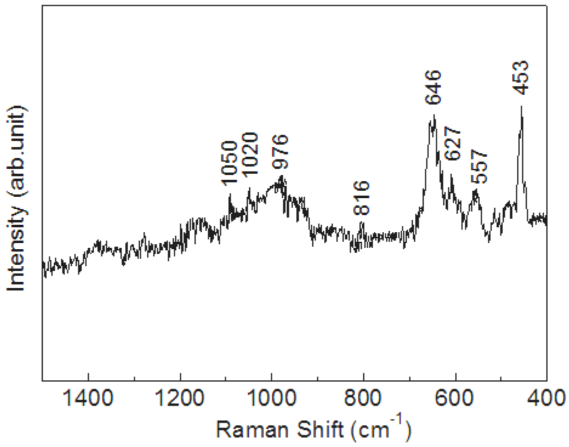

Figure S2. Raman spectra of the CHP-IP6 powers with various morphologies.

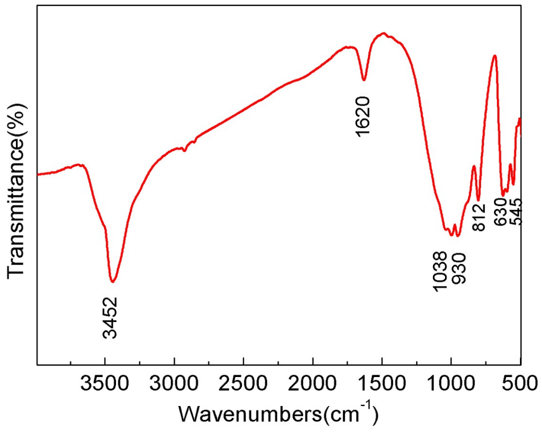

Figure S3. FTIR spectra of CHP-IP6.

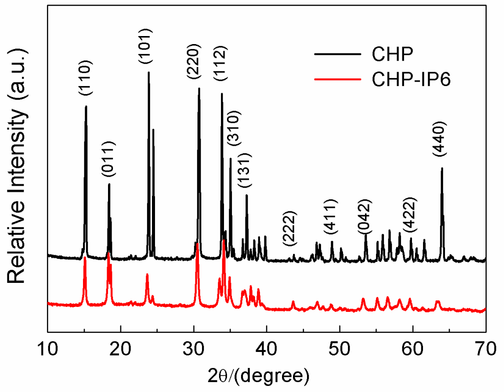

Figure S4. XRD patterns of CHP and CHP-IP6.

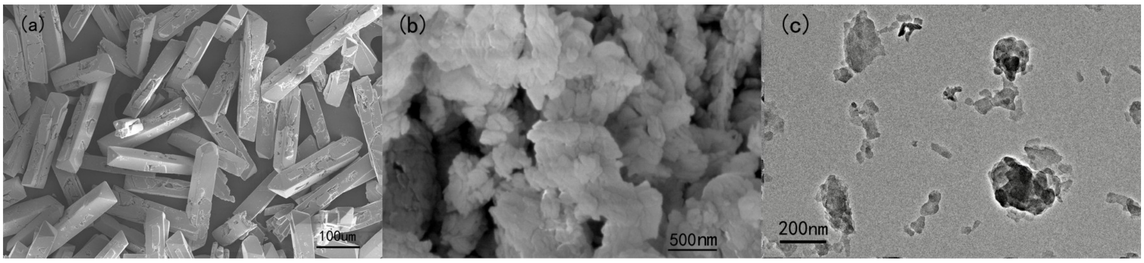

Figure S5. SEM images of (a) CHP, (b) CHP-IP6 and (c) TEM of CHP-IP6.

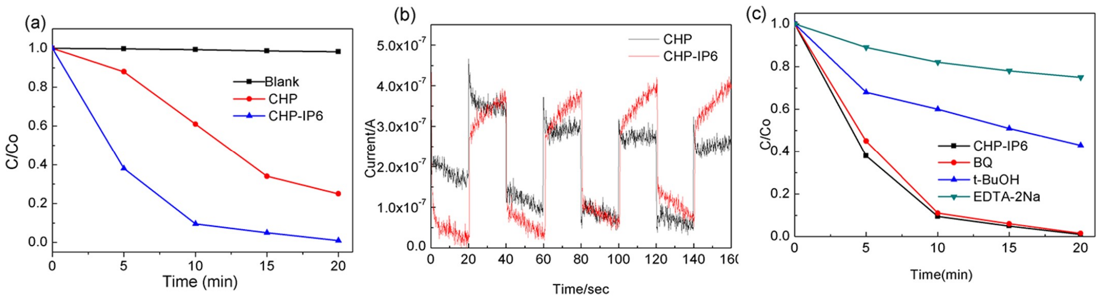

Figure S6. (**a**) Photocatalytic MB degradation under Vis-NIR irradiation; (**b**) transient photocurrent responses of CHP and CHP-IP6; (**c**) Fig. 7 effect of the different scavengers on RhB degradation for CHP-IP6 .

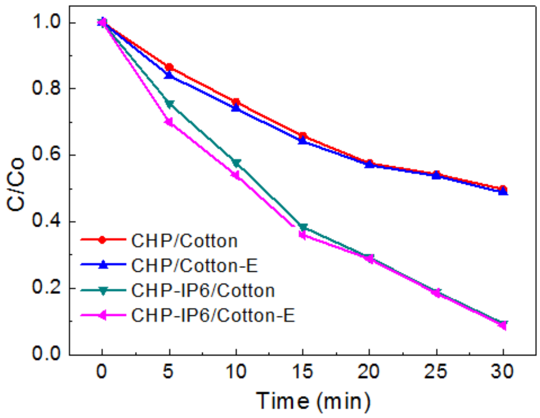

Figure S7. Photocatalytic MB under Vis-NIR (CHP-Cotton-E & HP-IP6-E tested after irradiation and 30min balance in the dark).

Table S1. The *K*/*S* values of the samples.

samples	K/S values After Different Irradiation Time				
	**0 h**	**3 h**	**6 h**	**9 h**	**12 h**
cotton fabric	9.38	9.24	9.02	8.87	8.66
CHP coated cotton fabirc	6.97	3.08	1.64	0.12	0.10
CHP-IP6 coated cotton fabirc	7.42	2.52	1.01	0.06	0.04

© 2019 by the authors. Licensee MDPI, Basel, Switzerland. This article is an open access article distributed under the terms and conditions of the Creative Commons Attribution (CC BY) license (http://creativecommons.org/licenses/by/4.0/).
